# Temporal Uncertainty and Temporal Estimation Errors Affect Insular Activity and the Frontostriatal Indirect Pathway during Action Update: A Predictive Coding Study

**DOI:** 10.3389/fnhum.2016.00276

**Published:** 2016-06-27

**Authors:** Roberto Limongi, Francisco J. Pérez, Cristián Modroño, José L. González-Mora

**Affiliations:** ^1^College of Medicine, Valparaíso UniversityValparaíso, Chile; ^2^Department of Psychology, Diego Portales UniversitySantiago, Chile; ^3^Department of Physiology, Faculty of Medicine, Universidad de la LagunaSan Cristobal de la Laguna, Spain

**Keywords:** temporal prediction, predictive coding, dynamic causal modeling, action update, prediction errors

## Abstract

Action update, substituting a prepotent behavior with a new action, allows the organism to counteract surprising environmental demands. However, action update fails when the organism is uncertain about when to release the substituting behavior, when it faces temporal uncertainty. Predictive coding states that accurate perception demands minimization of precise prediction errors. Activity of the right anterior insula (rAI) is associated with temporal uncertainty. Therefore, we hypothesize that temporal uncertainty during action update would cause the AI to decrease the sensitivity to ascending prediction errors. Moreover, action update requires response inhibition which recruits the frontostriatal indirect pathway associated with motor control. Therefore, we also hypothesize that temporal estimation errors modulate frontostriatal connections. To test these hypotheses, we collected fMRI data when participants performed an action-update paradigm within the context of temporal estimation. We fit dynamic causal models to the imaging data. Competing models comprised the inferior occipital gyrus (IOG), right supramarginal gyrus (rSMG), rAI, right presupplementary motor area (rPreSMA), and the right striatum (rSTR). The winning model showed that temporal uncertainty drove activity into the rAI and decreased insular sensitivity to ascending prediction errors, as shown by weak connectivity strength of rSMG→rAI connections. Moreover, temporal estimation errors weakened rPreSMA→rSTR connections and also modulated rAI→rSTR connections, causing the disruption of action update. Results provide information about the neurophysiological implementation of the so-called horse-race model of action control. We suggest that, contrary to what might be believed, unsuccessful action update could be a homeostatic process that represents a Bayes optimal encoding of uncertainty.

## Introduction

Action update, the ability to replace an ongoing behavior with a new action, enables us to navigate a volatile environment. In the cognitive literature, action update is also referred to as response reengagement (Boecker et al., 2011, 2013) or task switching (Limongi et al., 2015). In general, the stop-change paradigm (Logan, 1982, 1983) has been used to unveil the cognitive processes that mediate action update. In the stop-change task, two stimuli (S1 and S2) are presented in sequence. The participant must respond to S1 as quickly as possible. But, if S2 appears a few milliseconds after S1 the participant must substitute the S1-associated response with the response associated with S2. The probability of succeeding at substituting the S1-associated response decreases as the change signal delay (CSD, the delay of the S2 onset relative to the S1 onset) increases.

Recently, Limongi et al. (2015) reported that, after controlling for the effect of CSD, action update accuracy decreases if the organism fails to predict *when* to execute the new action (i.e., during inaccurate time estimation). Specifically, participants were instructed to prepare a response that had to be executed after the *implicit* estimation of a time interval, the covert estimation of time which is necessary to accomplish the task (Piras and Coull, 2011). On some trials, before the onset of the to-be estimated time interval, a signal cued participants to substitute the prepared response with a new response. Limongi et al. (2015) found that action update accuracy (referred to as task-switching performance accuracy) decreased as a function of both the estimated time interval and the time estimation error (TEE), referred to as temporal prediction error.

Behavioral studies have suggested that response inhibition is an essential stage during action update (Verbruggen et al., 2008), and neurophysiological studies suggest that response inhibition recruits activity of the indirect frontostriatal pathway associated with motor control (Ray Li et al., 2008; Duann et al., 2009; Zandbelt and Vink, 2010; Jahfari et al., 2011, 2012; Cai et al., 2012, 2014; Freeze et al., 2013; Obeso et al., 2013; Watanabe et al., 2015). If response inhibition is crucial for successful action update and both temporal uncertainty and TEEs affect action update, then both temporal uncertainty and TEEs should influence the indirect frontostriatal pathway associated with response inhibition.

In the indirect frontostriatal pathway, the right presupplementary motor area (rPreSMA) projects to *indirect* medium spiny neurons (iMSN) of the right striatum (rSTR). These neurons constitute a subpopulation of MSN that project to the external segment of the globus pallidus (Wall et al., 2013), leading to response inhibition. Another frontal region, the right anterior insula (rAI) also projects to iMSN. However, unlike the rPreSMA, the rAI is strongly associated with both unsuccessful response inhibition (Cai et al., 2014) and uncertainty (Volz et al., 2003; Hsu et al., 2005; Grinband et al., 2006; Platt and Huettel, 2008; Schultz et al., 2008; Sarinopoulos et al., 2010; Mushtaq et al., 2011; Venkatraman and Huettel, 2012; Grupe and Nitschke, 2013; Limongi et al., 2013; Payzan-Lenestour et al., 2013; Yoshida et al., 2013; Ghahremani et al., 2015). These empirical works suggest that the rAI plays a central role when temporal uncertainty and TEEs induce unsuccessful action update.

The hypothesis of a functional relationship between the rAI and unsuccessful action update also resides on theoretical grounds. Based on a predictive coding perspective (Rao and Ballard, 1999; Friston and Kiebel, 2009; Huang and Rao, 2011), it has been recently proposed that the effect of temporal uncertainty on action update is caused by *imprecise* message passing from sensory to motor areas (Limongi et al., 2015). In predictive coding, the rAI features a hub for sensorimotor integration (Gu et al., 2013; Limongi et al., 2014, 2015) because it links sensory and associative areas such as the occipital and parietal cortices with motor areas such as the rPreSMA and rSTR. The influence of temporal uncertainty on the rAI would be to decrease the insular sensitivity to ascending prediction errors because, in predictive coding, precision of prediction errors is thought to correspond to their synaptic gain (Feldman and Friston, 2010).

Based upon the above empirical and theoretical bases, we hypothesize that the disruption effect of temporal uncertainty on the indirect pathway occurs via direct influence of temporal uncertainty on the rAI. Furthermore, because estimation errors have been shown to modulate cortico-striatal coupling (den Ouden et al., 2010; Parka et al., 2012), we also hypothesize that the disruption effect of TEEs on action update occurs via modulatory influences on either rPreSMA→rSTR or rAI→rSTR connections. Moreover, this effect could vary depending upon the magnitude of the temporal gap on the rAI. In this work, we used event-related fMRI and dynamic causal modeling (DCM) to investigate these hypotheses. We asked participants to perform action updates within the context of implicit time estimations with different levels of temporal uncertainty induced by the magnitude of the target temporal interval —referred to as temporal gap (Limongi et al., 2015). We used temporal uncertainty and TEEs as parametric explanatory variables in a general linear model (GLM) fitted to the fMRI data. Following, we used DCM to test for driving and modulatory effects of temporal uncertainty and TEEs on the rAI activity and frontostriatal connections associated with the indirect pathway.

## Materials and Methods

### Participants

Sixteen right-handed students (9 females, *M* age = 22.4 years SD = 5.51) from Universidad de la Laguna signed an informed consent form and participated in the study. The ethics committee of Universidad de la Laguna approved the study.

### Task and Stimuli

**Figure 1:**
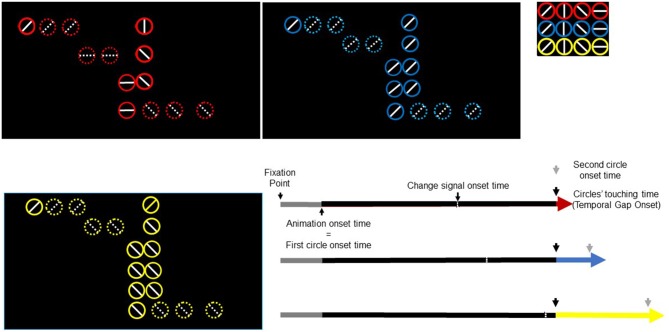
**Trial timeline for three task conditions and three temporal gaps.** Black rectangles depict three temporal-gap conditions. Colored circles signal the temporal gap (red, 0 ms; blue, 300 ms; yellow, 600 ms). Inner diameter lines take any of four possible orientations (shown in the upper right small rectangle). In this figure, the circles in red exemplify the false-alarm task condition whereas the circles in blue and yellow exemplify the no-change and change-task conditions respectively. Three extended arrows (bottom right) depict events times. A fixation point lasted 500 ms (gray). The animation onset time and the first circle onset time occurred simultaneously. The first circle moved towards the right-most circle (black part of the arrow) and stopped at the edge of the second circle. The red/blue/yellow segment of the arrow represents the temporal gap whose offset signals the second circle onset. The change-signal onset time (CSO) occurred 640 ms before the circles’ touching time.

We used a modified version of a task used in Limongi et al. (2015). Participants performed temporal predictions of two circles colliding like billiards balls (Figure 1). A trial comprised two events: fixation point (500 ms) and visual animation (3100 ms). At the animation onset, two colored (red, blue, or yellow) circles (1.30° of visual angle in diameter) with a white inner line (horizontal, vertical, left-diagonal, or right-diagonal) simultaneously appeared on the left and center of a screen with a black background. Then, the left-most circle (first circle in Figure 1) moved to the center of the screen at a constant speed (17.32°/s) until it stopped 1000 ms later at the edge of the second circle. After a temporal gap (0, 300, or 600 ms) the right-most circle (second circle in Figure 1) began moving to the right. Circles’ colors informed participants about the magnitude of the temporal gap to be estimated (red, 0 ms; blue, 300 ms; and yellow, 600 ms). Participants pressed a response button when they predicted the second circle onset. They pressed one button if, when they responded, circles’ inner lines were alike (e.g., “vertical” and “vertical” for the first and second circle respectively) and another button if, when they responded, circles’ inner lines were different (e.g., “horizontal” and “diagonal”). Eight participants used the index finger to respond “same” (thumb to respond “different”) and eight participants used the thumb to respond “same” (index to respond “different”). Visibility of both circles remained until the end of the trial (3600 ms after the trial onset). The time interval between the animation onset and the gap onset was constant across trials and conditions.

We constructed a 3 × 3 factorial design: temporal gaps (with three levels: 0, 300, 600 ms) times tasks (with three levels: change, no change, and false alarm). The change task was our event of interest whereas the false-alarm and the no-change tasks were included as control tasks to prevent the participants from anticipating an action-update demand. Moreover, the false-alarm task would also prevent participants from performing action update relying solely on the sensory salience caused by the change in the orientation of the inner lines, without contrasting their relational value (e.g., from same to different).

Each task condition comprised 33% of trials. In the change and false-alarm conditions, the circles’ inner lines changed 640 ms before the second circle onset. For example, if at the animation onset the inner lines were “vertical” and “vertical” (for the first and second circle respectively) they changed to “diagonal” and “horizontal”. We will refer to the change time of lines as the change-signal onset time (CSO). In the false-alarm condition, lines change in orientation, but the relational value remained. For example, if the initial lines were “horizontal” and “vertical” (for the first and second circle respectively) they could change to “left-diagonal” and “horizontal”. Notice that despite this change, lines’ relational value (i.e., different) was the same. In the no-change condition, lines did not change. The stimulus delivery program randomly chose the combination of lines. The program also randomly varied the initial positions of circles in the horizontal axis. However, the initial distance between circles was constant across trials. Figure 1 shows the sequence of events in a single trial.

Each participant performed at least one 36-trial familiarization block outside the scanner and received feedback from the experimenter. The experimenter carefully instructed the participant to “predict *when* the second circle would move rather than to react upon the second circle onset”. Within the scanner, participants executed 18 practice trials. They then performed ten 72-trial blocks divided into three sessions (sessions one and two, three blocks; session three, four blocks). Between two blocks of trials, participants rested 20 s and were encouraged to relax. In total, each participant performed 720 trials (240 trials/condition). After finishing the functional sessions, a standard 3D T1 image was acquired. During the acquisition, participants performed 100 trials of a warned reaction-time (RT) task. Reaction time data were collected to define a subject-wise response validity criterion, explained below.

Behavioral dependent variables of interest were both the response accuracy (regarding the relational value of circles’ inner lines) and the absolute TEE (|response time—second circle onset time|; Young et al., 2005; Limongi et al., 2013, 2015). Notice that the absolute values of TEEs are related to their squared values. This means that absolute values can be taken as a proxy for the precision (inverse variance) of behavioral response times. Regardless of the magnitude of the temporal gap, participants sometimes made predictions before and after the second circle onset (early and late predictions respectively). We used the mean RT computed from the post-experiment RT task (subject-wise) to set a validity criterion *for* *late predictions*.

### fMRI Data Acquisition

Axially oriented functional images were obtained by a 3T Signa HD MR scanner (General Electric Healthcare, Waukesha, WI, USA) using an echo-planar-imaging gradient-echo sequence and an 8-channel head coil (repetition time (TR) = 2500 ms, echo time (TE) = 36 ms, flip angle = 90°, matrix size = 64 × 64 pixels, 36 slices, 4 × 4 mm in plane resolution, spacing between slices = 4 mm, slice thickness = 4 mm plus 0 mm interslice gap, sequential acquisition).

Slices were aligned to the anterior–posterior commissure (AC–PC) line and covered the whole brain. Functional scanning was preceded by 18 s of dummy scans to ensure tissue steady-state magnetization. Images were taken during three different runs for every participant (runs 1 and 2: 330 volumes; run 3: 443 volumes). High resolution sagittally oriented anatomical images were also collected for anatomical reference. A 3D fast spoiled-gradient-recalled pulse sequence was obtained (TR = 8 ms, TE = 1 ms, flip angle = 12°, matrix size = 256 × 256 pixels, 0.98 × 0.98 mm in plane resolution, spacing between slices = 1 mm, slice thickness = 1 mm).

### Behavioral Data Analysis

All early predictions (responses before collisions) were considered valid responses. However, only late predictions (responses after the second circle onset) whose TEEs were smaller than the subject’s mean RT—computed from data collected during the post-scanning warned-RT task—were deemed valid. We used this criterion to exclude responses that could be reactions to the second circle onset rather than true predictions. With data from valid trials, we verified that absolute TEEs increased linearly as a function of the temporal gap (Young et al., 2005; Limongi et al., 2013, 2015). To this aim, we regressed absolute TEEs on temporal gaps and included participants as random effects in a simple linear mixed-effects model.

We then aimed at replicating results reported in Limongi et al. (2015) by fitting four mixed-effects linear models to accuracy data. For this analysis, absolute TEEs were coded in terms of Vincentiles (Balota and Yap, 2011). Large Vincentiles represented large TEEs. Model 1a comprised the main effect of task, the main effect of absolute TEE, and the Task × Absolute TEE interaction. Model 2a included all effects of model 1a, the main effect of temporal gap, and the Temporal Gap × Task interaction. Two additional models (models 1b and 2b) included the Task × CSO interaction as a possible predictor of the action-update performance accuracy (Verbruggen et al., 2008). Model 3 only included the main effect of task, the main effect of CSO, and the Task × CSO interaction. In addition to these four models, we also tested for the effect of TEE × Temporal Gap interaction. As stated in the introduction, it is possible that the effect of absolute TEEs on frontostriatal connections varies with the effect of temporal gaps on the rAI. Therefore, a fifth model (model 4) comprised all predictors of model 2a and the TEE × Temporal Gap interaction. We selected the best model based upon the models’ corrected Akaike information criterion numbers (AIC_c_). AIC_c_ corrects AIC for sample size (*n*) *n* < 40. We report Akaike weights (Wagenmakers and Farrell, 2004) and *F* statistics for a more intuitive understanding about the relative merits of models and a classical interpretation of fixed effects respectively.

### fMRI Data Analysis

All data analyses and modeling were performed on SPM12 (Wellcome Trust Centre for Neuroimaging, London, UK). Anatomical images were manually reoriented, matching the *y* axis to the AC-PC line and setting the origin approximately 3 mm bellow the AC. All functional images were automatically reoriented to match these coordinates. Functional images were realigned and coregistered with structural scans. Structural scans were segmented into white and gray matters. A group-specific template was created via the DARTEL utility followed by non-linear image registration procedure and smoothed with no modulation and a Gaussian full width at half maximum (FWHM) of 8 × 8 × 8. With the resulting template, functional images were normalized to the Montreal Neurological Institute (MNI) space.

#### GLM and Classical Inference

A GLM was fitted to fMRI data. The model comprised three regressors representing three task levels (no-change, false-alarm, and change). Each regressor was parametrically modulated first by the temporal gap and second by the absolute TEE. Notice that the absolute TEE was the second parametric modulator, accounting for the variance not explained away by the temporal gap. Six head movement parameters were also included as regressors of no interest. Events were time locked to the second circle onset. At a subject level, we created images per each task condition and each parametric modulator (nine images in total).

Random effects analysis was performed as follows. First, we tested for the effect of action update after accounting for the effects of both temporal gap and absolute TEE. To this aim, we searched for activity in the change vs. baseline contrast. Second, we focused on performance in terms of the effect of parametric modulators during the change task by searching for activity in the Change Task × Temporal Gap and Change Task × Absolute TEE interactions. In concrete, we searched for activity in five regions of interest (ROIs) whose (performance-specific) coupling was then specified using DCM. Based on our hypotheses, we searched for activity in the rAI, the right inferior occipital gyrus (rIOG), the right supramarginal gyrus (rSMG), the rPreSMA, and the rSTR. All five regions participate in response inhibition tasks (Sharp et al., 2010; van Gaal et al., 2010; Jahfari et al., 2011; Berkman et al., 2012; Mahmood et al., 2013; Majid et al., 2013). We created a single 5-region mask. Each region consisted in a sphere of 10-mm radius centered at the MNI coordinates reported in a recent meta-analysis by Cai et al. (2014) (rIOG, *X* = 48, *Y* = −74, *Z* = −12; rSMG, *X* = 52, *Y* = −42, *Z* = 38; rAI, *X* = 38, *Y* = 20, *Z* = −4; rSTR, *X* = 14, *Y* = 8, *Z* = 6) and by Jahfari et al. (2012) (rPreSMA, *X* = 9, *Y* = 24, *Z* = 50).

In addition to the ROI analysis, we performed two whole-brain exploratory analyses. We specifically looked at condition-specific and parametric modulator-specific effects as determined by the experimental design. First, we tested for positive effects of each task condition contrasted against the implicit baseline. Second, we tested for positive and negative effects of each parametric modulator across all three task conditions.

##### Criteria for statistical significance and precise anatomical location

For the ROI analysis, activity yielded by a search within the 5-region mask was reported as statistically significant if it survived the familywise error (FWE) correction of *p* < 0.05, at a voxel level. Maxima were verified on the group-specific DARTEL-generated template. For the whole-brain analyses, we considered activity as statistically significant if it survived the FWE correction of *p* < 0.05, also at a voxel level.

#### Effective Connectivity Analysis (DCMs)

##### Time series extraction

Time series (first eigenvariate) of a target ROI was extracted if the subject’s activation maximum survived an uncorrected threshold of *p* < 0.05, if it was located within a sphere (with a radius of 8 mm) centered on the group’s maximum, and if the activation maximum was located in the gray matter of the subject’s anatomical scan. After applying these criteria, data from two subjects were excluded from the DCM analysis. We used the Change × Temporal Gap interaction contrast to identify participant’s maxima in the rIOG, rSMG, rPreSMA, and rAI whereas the Change × TEE interaction contrast was used to identify participant’s maxima in the rSTR. We extracted the times series (adjusted for the effects of interest, using the *F* contrast comprising all conditions) of all voxels within a sphere (with a radius of 5 mm) centered at the participant’s maximum. Regarding each extracted time series, we computed the mean proportion and standard deviation of explained variance (rAI, *M* = 0.88, SD = 0.08; rIOG, *M* = 0.87, SD = 0.06; rSMG, *M* = 0.87, SD = 0.05; rPreSMA, *M* = 0.86, SD = 0.07; and rSTR, *M* = 0.81, SD = 0.05).

##### DCM specification

We specified two-state bilinear DCMs (Marreiros et al., 2008; Bastos et al., 2012) based upon predictive coding assumptions (i.e., hierarchical arrangement and bidirectional connections), *a priori* information about the nature of the task, functional and anatomical connectivity constraints, and our specific hypotheses (Stephan et al., 2010). We used a heuristic (greedy) search to identify the best DCM or architecture that could explain our data and test our hypotheses. This search comprised two steps. First, we considered 24 models with various combinations of driving effects of temporal gaps and modulatory effects of TEEs. Having identified the best combination, we then considered four models that represented the best model, two reduced models, and one non-nested (NN) model. In detail, endogenous connections, driving/modulatory inputs, and model spaces were specified as follows.

##### Endogenous connections

All connections were assumed bidirectional. The rIOG connected with the rSMG (Joshi et al., 2010). The rSMG connected with the rAI (Cauda et al., 2011; Cai et al., 2014). The rAI connected with both the rPreSMA (Zhang et al., 2012) and the rSTR (Chikama et al., 1997; Cauda et al., 2011). Finally, the rPreSMA connected with the rSTR (Zhang et al., 2012).

##### Driving and modulatory inputs

In our basic model, the stimuli of the change task entered the rIOG. Behaviorally, the probability of changing an ongoing behavior (action update) decreases as both temporal gaps and absolute TEEs increase (Limongi et al., 2015). Because we hypothesize that the uncertainty induced by temporal gaps drives activity into the rAI, our basic model included temporal gaps as driving inputs into this region. Moreover, because we also hypothesize that absolute TEEs modulate connections from either the rAI or the rPreSMA to the rSTR, we included absolute TEEs as modulatory inputs to these connections.

##### Basic model space

**Figure 2:**
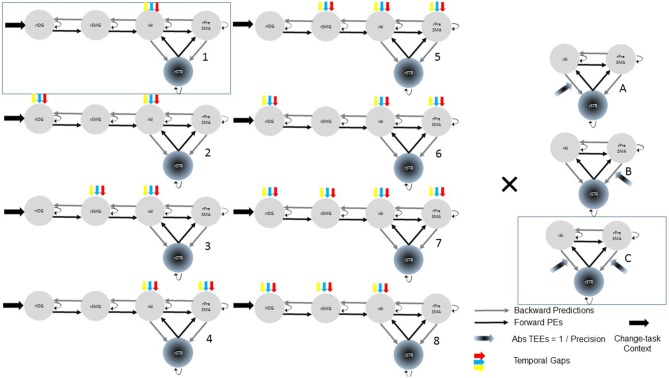
**Model space with 24 (8 × 3) possible models representing our hypotheses.** In all models, all the trials of the change-task condition entered the rIOG and temporal gaps drove activity at least into the rAI. TEEs modulated at least one corticostriatal connection. Combinations of driving inputs are represented with numbers (1–8). Combinations of modulatory inputs are represented with letters (A–C). Each single model is represented by one number and one letter (e.g., A3). Bayesian model selection provided support to the model 1C (within the rectangle).

Although we hypothesize that temporal gaps drive activity into the rAI, driving inputs associated with temporal gaps could also affect the rSMG, rIOG, or the rPreSMA. Similarly, modulations exerted by TEEs could affect either rPreSMA→rSTR or rAI→rSTR connections. To remove these uncertainties, we constructed 24 models with systematic combinations of driving (eight combinations, 1–8) and modulatory (three combinations; A, B, C) inputs (Figure 2). Within this model space, we searched for the best model that represented our hypotheses.

##### Model space with alternative reduced and non-nested models

**Figure 3:**
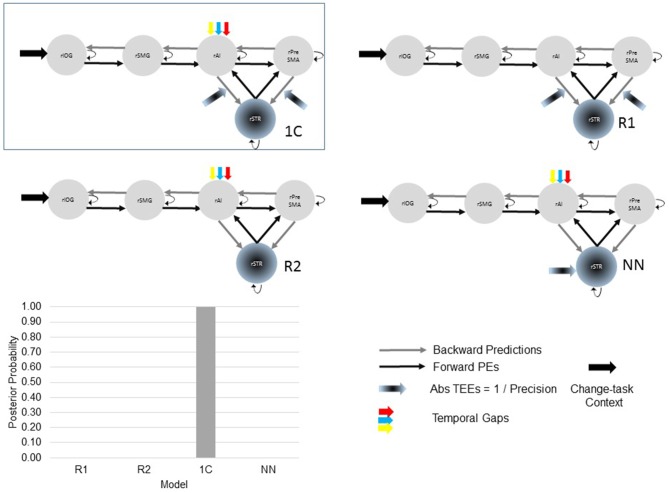
**The optimal (winning) model and three competing models testing our hypotheses.** Two of the alternative (reduced) models (R1 and R2) were constructed by removing, from the winning (full) model, driving and modulatory inputs defined in our hypotheses. The other, non-nested (NN), model was defined upon previous works. Bayesian model selection provided support to the winning model 1C (PP > 0.99). The PPs of the three alternative models are close to 0.

After selecting the best model within the 24-model space, we defined a new model space with the winning model, two reduced models and one NN model (Figure 3). Reduced models were constructed by selectively removing inputs representing our hypotheses. In the reduced model 1 (R1), we removed driving inputs into the rAI. In the reduced model 2 (R2), we removed modulations of both the rPreSMA→rSTR and the rAI→rSTR connections. Finally, the NN model was inspired by previous results showing that behavioral prediction errors affect striatal activity (den Ouden et al., 2009, 2010; Limongi et al., 2013). Specifically, it is possible that activity in the rSTR is affected by the direct influence of TEEs rather than by the modulation of frontostriatal connections. Therefore, in the NN model TEEs drove activity into the rSTR.

##### Model comparison and selection, and parameters inference

We relied on fixed-effects Bayesian model selection (Stephan et al., 2009, 2010) and selected the model with the largest posterior probability (PP). After selecting the optimal model and testing it against the alternative architectures, we performed inferences about the physiological effects of temporal gaps and TEEs on regions and connections. To this aim, we computed Bayesian averages of parameter values (Kasess et al., 2010). For each contrast of interest, we relied upon a PP = 0.90 as a confidence threshold.

## Results

### Behavioral Results

**Figure 4:**
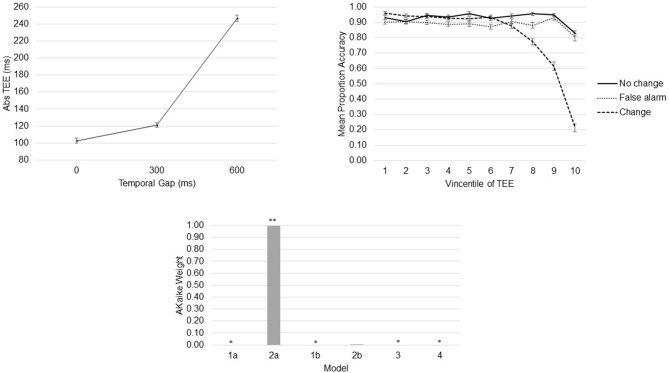
**Behavioral Results.** Absolute time estimation error (TEE) increased as a function of the temporal gap (top left). Task-update performance decreased as a function of the absolute TEE (top right). Akaike weights provide evidence to support a model with independent effects of temporal gaps and absolute TEEs on action-update performance (bottom). *Akaike weight close to 0. **Akaike weight close to 1.

Participants performed the task as instructed (the mean percentage of valid trials was 98.08, SD = 2.25). As expected, and replicating previous results (Young et al., 2005; Limongi et al., 2013, 2015), the linear model shows that TEEs increased as a function of the temporal gap (*β* = 0.24, *SE* = 0.006), *F*_(1,11170)_ = 1351.7, *p* < 0.0001 (Figure 4). Also replicating previous results (Limongi et al., 2015), the model comparison strategy yielded model 2a as the winning model (AIC_c_model2a_ = 4816). AIC_c_ numbers and Akaike weights (Figure 4) show that all other models poorly performed (AIC_c_model1a_ = 4877, AIC_c_model1b_ = 4828, AIC_c_model2b_ = 4829, AIC_c_model3_ = 5533, AIC_c_model4_ = 4830). Table 1 shows the winning model’s parameter estimates. Clearly, action-update performance accuracy decreased as a function of both the temporal gap and the absolute TEE (Figure 4 and Table 1). Of relevance for our DCM hypotheses is the fact that both effects were orthogonal, as indexed by low variance inflation factors (VIF, Table 1). Moreover, behavioral data show that the effect of TEEs did not change with the magnitude of the temporal gap, as shown by the poor performance of model 4. From a classical inference perspective, *F* tests also confirm the expected effects; main effect of task, *F*_(2,11162)_ = 117.57, *p* < 0.0001; main effect of gap, *F*_(1,11163)_ = 49.52, *p* < 0.0001; main effect of Vincentile, *F*_(1,11162)_ = 497.43, *p* < 0.0001; Task × Gap interaction, *F*_(2,11162)_ = 43.02, *p* < 0.0001; and Task × Vincentile interaction, *F*_(2,11163)_ = 146.30, *p* < 0.0001).

**Table 1:** **Parameter estimates of the linear mixed-effects model**.

Predictor	Estimate	SE	DF	*t* Ratio	*P*	Lower 95%	Upper 95%	VIF
Intercept	0.9874	0.0205	18	48.09	<0.0001	0.9442	1.0306	
Task [false alarm]	0.0064	0.0040	11162	1.61	0.1082	−0.0014	0.0143	1.35
Task [change]	−0.0565	0.0040	11162	−14.12	<0.0001	−0.0644	−0.0487	1.35
Vincentile	−0.0245	0.0011	11162	−22.30	<0.0001	−0.0266	−0.0223	1.25
Task [false alarm] × (Vincentile − 5.49437)	0.0164	0.0015	11163	10.60	<0.0001	0.0134	0.0195	1.68
Task [change] × (Vincentile − 5.49437)	−0.0262	0.0015	11162	−16.90	<0.0001	−0.0292	−0.0231	1.68
Temporal Gap	0.0001	<0.0001	11163	7.04	<0.0001	0.0001	0.0001	1.23
(temporal gap − 306.329) × Task [false alarm]	0.0001	<0.0001	11162	3.14	0.0017	0	0.0001	1.57
(temporal gap − 306.329) × Task [change]	−0.0002	<0.0001	11162	−9.21	<0.0001	−0.0002	−0.0001	1.57

*Vincentile and temporal gap values are mean centered*.

### fMRI Results

**Figure 5:**
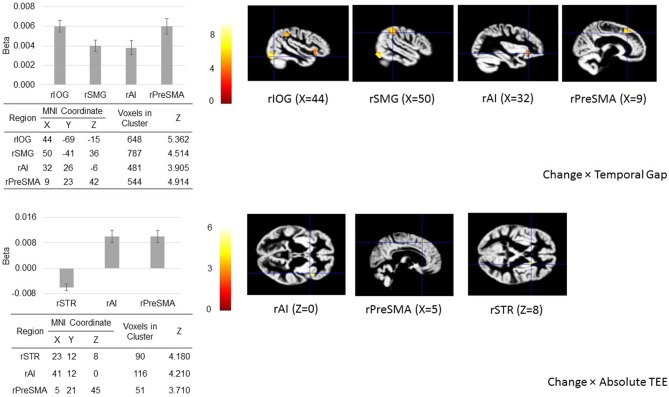
**Regions of interest (ROIs) analysis.** When action update was demanded (in the change-task condition), temporal gaps and absolute TEEs produced changes in the hemodynamic response of our* a priori* defined ROIs located in sensory (right inferior occipital gyrus, rIOG), associative (right supramarginal gyrus, rSMG), frontal (right presupplementary motor area, rPreSMA; right anterior insula, rAI), and striatal areas (right striatum, rSTR) associated with both sensorimotor integration and response inhibition. The figure shows regional peak activities overlaid on the group-specific anatomical template.

The ROI analysis shows that when action update was demanded, activity in the rIOG, rSMG, rAI, and rPreSMA increased as a function of the temporal gap, and activity in the rAI and rPreSMA increased as function of the absolute TEE. Crucially, rSTR activity decreased as the absolute TEE increased (Figure 5).

The first whole-brain analysis reveals that, compared with the implicit baseline, all three task conditions recruited the right fusiform gyrus and the left SMG. Furthermore, both the false-alarm and the no-change conditions recruited the right medial-frontal gyrus (supplementary motor area). Table 2 shows the complete list of peak activations. The second whole-brain analysis reveals that regardless of the task, uncertainty induced by temporal gaps increased activity in the visual, associative, and medial-frontal areas whereas absolute TEEs decreased activity in the striatum (Table 2).

**Table 2:** **Whole brain activations of task conditions and parametric modulations—collapsed across conditions**.

Condition	Side	Region	MNI coordinates			Voxels	*Z*-score
			*X*	*Y*	*Z*
Change	Right	Fusiform gyrus	50	−71	−11	57	5.27
	Left	Supramarginal gyrus	−47	−42	42	28	4.87
No change	Right	Fusiform gyrus	50	−72	−12	46	5.20
	Right	Supp. motor area	9	2	63	1	4.82
	Left	Supramarginal gyrus	−47	−42	42	19	4.81
False alarm	Right	Fusiform gyrus	50	−71	−12	113	5.64
	Left	Supramarginal gyrus	−47	−42	42	115	5.03
	Right	Supp. motor area	9	2	63	2	4.78
Temporal gap	Right	fusiform gyrus	48	−69	−12	1081	6.16
	Right	Fusiform gyrus	35	−66	−15		5.29
	Right	Inferior occipital gyrus	30	−90	−6		5.29
	Right	Cingulate gyrus	11	18	41	521	5.83
	Right	Supp. motor area	2	18	48		5.59
	Right	Supp. motor area	2	8	50		4.78
	Right	Pars opercularis	50	5	17	14	4.88
	Left	Fusiform gyrus	−47	−69	−14	136	5.46
	Left	Insula	−30	23	−3	21	5.01
	Left	Inferior occipital gyrus	−29	−87	−11	45	4.97
	Left	Insula	−29	23	5	1	4.78
	Right	Cerebellum (declive)	29	−71	−29	1	4.77
	Left	Supp. motor area	−9	6	45	7	4.85
	Right	Supp. motor area	5	6	51	1	4.77
TEE*	Left	putamen	−27	−3	−10.5	18	4.84
			−27	−10.5	1.5	2	4.68

*Deactivation.

### Effective Connectivity

**Figure 6:**
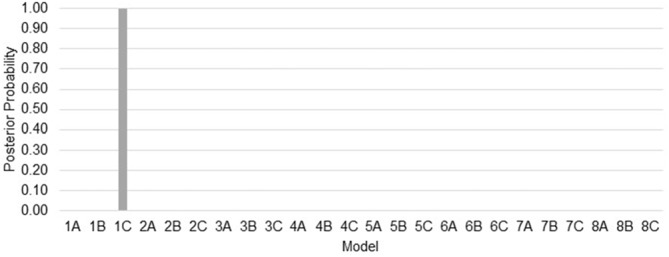
**Posterior probabilities of 24 models compared via Bayesian model selection.** The posterior probability (PP) of model 1C (the winning model) is close to 1 whereas the PPs of the other models are close to 0.

Bayesian model selection yielded model 1C as the winning model (PP > 0.99, Figure 6). Model 1C reveals that temporal gap drives activity only in the rAI whereas absolute TEEs modulate both rPreSMA→rSTR and rAI→rSTR connections. Moreover, this optimal model surpassed competing alternative models (PP > 0.99, Figure 3).

**Figure 7:**
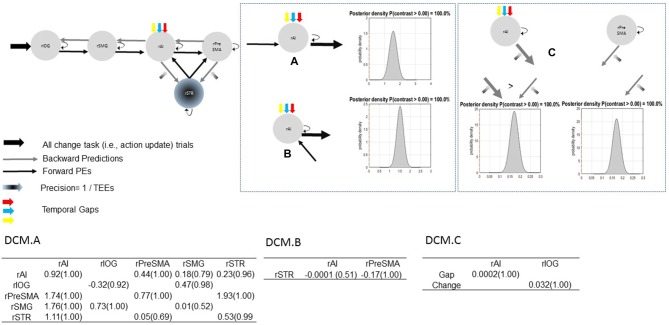
**Parameter estimates and PPs (yielded by Bayesian parameter averaging) of the winning model.** Tables within the figure show parameter estimates (PPs in parentheses); dynamic causal modeling (DCM) DCM.A, matrix of endogenous connections; DCM.B, matrix of modulatory inputs; DCM.C, matrix of driving inputs. Parameters are not exponentiated. The winning model (top left). Contrast between strengths of rSMG→rAI and rAI→rPreSMA connections (A). Contrast between strengths of rSTR→rAI and rAI→rPreSMA connections (B). Contrast between modulatory effects of absolute TEEs on rAI→rSTR and on rPreSMA→rSTR connections (C). Arrow thickness depicts the relative parameter value or relative connection-strength value. Density plot represents the posterior density yielded by the contrast of interest.

Bayesian parameter averaging provides evidence in support of our hypotheses. Figure 7 shows parameter estimates with PPs and posterior densities of the contrasts of interest. First, uncertainty induced by increasing temporal gaps drove activity into the rAI (PP = 1.00). As a result, the rAI decreased sensitivity to ascending (forward) connections from the rSMG and from the rSTR when contrasted against rAI→rPreSMA connections (PP = 1.00). Moreover, TEEs negatively modulated rPreSMA→rSTR connections (PP = 1.00). Notice that the PP of the parameter estimate representing the modulatory effect of TEEs on rAI→rSTR connections (PP = 0.53) does not provide information to infer about its relative effect. This is, although at the level of the model structure (Stephan et al., 2010) we are confident to conclude that there is a modulatory effect of TEEs on rAI→rSTR connections, we do not have sufficient evidence to adjudicate between positive and negative effects. However, the PP yielded by the contrast between both modulatory effects (i.e., rPreSMA→rSTR vs. rAI→rSTR, PP = 1.00) provides evidence to confidently infer that the dampening effect of absolute TEEs was stronger on rPreSMA→rSTR connections than on rAI→rSTR connections.

## Discussion

We tested the hypotheses that when participants perform action update within the context of temporal estimation, uncertainty induced by temporal gaps affects the rAI sensitivity to ascending prediction errors, and TEEs modulate corticostriatal connections. These changes in the effective connectivity of the network cause action-update performance accuracy to decrease. At a behavioral level, we replicated previous results (Limongi et al., 2015). At a neurophysiological level, the DCM analysis showed that temporal gaps influence the rAI, and TEEs influence rAI→rSTR and rPreSMA→rSTR connections. The analysis also showed that the action-update (change-task) context influences the visual cortex. Parameter estimates confirm the hypothesized decreased sensitivity of the rAI to ascending prediction errors. Parameter estimates also indicate that TEEs weaken more the rPreSMA→rSTR connections than the rAI→rSTR connections.

Activity in the rAI not only increases as a function of the uncertainty about *when* a stimulus occurs but also as a function of the uncertainty about *whether* the stimulus actually occurs. This has been specifically demonstrated by previous data showing that activity in the rAI increases as a function of the unsigned or absolute stimulus prediction error (Hu et al., 2015). Because these prediction errors index the uncertainty associated with stimulus occurrence, it appears that the rAI is especially sensitive to both temporal and non-temporal uncertainty during response selection (Ghahremani et al., 2015).

The effect of TEEs on rPreSMA→rSTR connections is consistent with previous data regarding stop signal expectations in the stop-signal task (Hu et al., 2015). When participants expect more a Stop signal on Go trials, prolonged reaction times are associated with increased activity in the rPreSMA. From a cognitive perspective, this is heuristically interpreted as an increase in predictions of an ensuing inhibition demand associated with strong rPreSMA→rSTR connections. This cognitive interpretation is fairly consistent with a predictive coding interpretation of the modulatory effect of TEEs. When TEEs are small, rPreSMA→rSTR connections are strong. This means that descending proprioceptive predictions increase, facilitating the ensuing prepotent response inhibition and, ultimately, action update. Interestingly, long RTs associated with failed predictions of stimulus occurrence and long response times associated with temporal predictions exert opposite effects on rPreSMA→rSTR influences.

In line with our results, other effective connectivity studies have shown changes in rPreSMA→rSTR connections during response inhibition. Using DCM, Rae et al. (2015) found non-linear modulatory effects of the right inferior frontal gyrus (rIFG) on rPreSMA→rSTR connections. Also with DCM, Li et al. (2014) reported that the integrity of rPreSMA→rSTR connections may be weaker in participants with behavioral disorders compared with control participants. Moreover, using Granger causality analysis Jahfari et al. (2011) showed that unsuccessful inhibitions would be more associated with rPreSMA→rSTR connections than with rIFG→rSTR connections. Expanding previous works showing that modulations of these connections occur during response inhibition, current results show that modulations of frontostriatal connections also occur during action update, in which response inhibition is an essential stage (Verbruggen et al., 2008).

Because action update implies response inhibition, our DCM analysis reveals new evidence in support of the thesis that corticostriatal connections implement a race between Go and Stop processes as defined in the so-called race model of acts of control (Logan and Cowan, 1984; Logan et al., 2014). The horse-race model states that Go and Stop processes independently run towards an execution threshold. The first process reaching the threshold wins the race. The current DCM model makes physiologically plausible these behavioral assumptions via simultaneous effects of TEEs on both the direct and the indirect pathways. This interpretation is consistent with single-cell recording data suggesting that the indirect/hyperdirect striatopallidal/striato-subthalamic pathways would implement the Stop process whereas the direct striatonigral pathway would implement the Go process (Schmidt et al., 2013; Noorani and Carpenter, 2014). Our interpretation is also consistent with a recent proposal that intrinsic striatal connections coordinate simultaneous activity of the indirect and direct pathways (Calabresi et al., 2014). Specifically, whereas the rPreSMA activates striatopallidal connections via iMSN, the rAI activates striatonigral neurons (rSTRn) via *direct* middle spiny neurons (dMSN)—a subpopulation of striatal neurons that project to rSTRn (Wall et al., 2013). Therefore, the rAI would facilitate the Go process, and the rPreSMA would facilitate the Stop process. Within this context, the winning DCM model predicts that the Go process wins the race against the Stop process because TEEs cause rPreSMA→rSTRp connections to be weaker than rAI→rSTRn connections.

Competition between rival processes is also present in the longstanding conflict monitoring model of cognitive control (Carter et al., 1998; Botvinick et al., 1999; Botvinick and Cohen, 2014). In the conflict monitoring model, the brain generates two signals associated with, for example, ongoing (i.e., Go) and substituting actions (i.e., Stop/Change). On error trials, the signal associated with the ongoing response overwhelms the competing signal, generating an implicit computation of error which is further monitored by the anterior cingulate cortex (ACC). The similarity between the conflict monitoring hypothesis about cognitive control and the current neurophysiological interpretation of the horse-race model of action control could motivate a future study. Specifically, it would be worth investigating whether there exists an error signal associated with the difference in strength between rPreSMA→rSTR and rAI→rSTR connections and whether such a signal is associated with an ACC-coordinated monitoring process.

From another theoretical perspective, Zarr and Brown (2016) recently proposed that inaccurate perception causes inaccurate performance. In line with this theoretical proposal, current results provide empirical evidence about how inaccurate perceptions prevent the organism from modifying ongoing behaviors. Predictive coding states that prediction errors that have not been minimized at lower levels of the cortical hierarchy are passed along to higher levels (Rao and Ballard, 1999; Bastos et al., 2012). Decreased sensitivity of the rAI to ascending prediction errors associated with the sensory processing of the change signal means that prediction errors were not minimized when the message passing cycle reached this region, being passed along to higher cortical levels. From the predictive coding perspective, the brain knows that this ascending (i.e., sensory) information associated with change signal is imprecise and relies on prior beliefs that the environment will not demand an action update. These prior beliefs would lead to the ensuing execution of the prepotent behavior (Limongi et al., 2015). In predictive coding terms, imprecise ascending prediction errors cause imprecise motor or proprioceptive predictions.

The above discussion motivates a broader interpretation consistent with a current theory of active inference, behavioral control, and homeostatic control systems (Friston et al., 2010, 2011; Adams et al., 2013; Clark, 2013; Shipp et al., 2013; Pezzulo et al., 2015). Our results suggest that inaccurate actions could be conceptualized as homeostatic sensorimotor responses. For example, reaching and grasping an object demand accurate visual estimates of the object’s location while the organism executes the reaching action. An inaccurate estimation of the visual target (i.e., sub-optimal Bayes estimates or sub-optimal minimization of perceptual prediction errors; Schwartenbeck et al., 2015) would cause the grasping maneuver to fail. However, the unsuccessful maneuver would pay off by allowing the minimization of free energy associated with the sub-optimal minimization of perceptual prediction errors because it appears that a conflict between signals associated with the ongoing response and signals associated with the new response increases free energy (Limongi et al., 2015). The organism would avoid this scenario by releasing free energy in terms of the minimization of proprioceptive (motor) prediction errors. This would be achieved via execution of the ongoing behavior.

To conclude, contrary to what might be believed, we speculate that unsuccessful action update may be an adaptive process that represents a Bayes optimal encoding of uncertainty or precision. Within the context of low temporal uncertainty (e.g., 0-ms gap), the precision of sensory prediction errors associated with the detection of the change (or action update) signal is attenuated at the level of the rAI; thereby enabling action update through reflexive responses to (precise) descending proprioceptive predictions. In contrast, within the context of high temporal uncertainty (e.g., 600-ms gap), the precision of sensory prediction errors is itself attenuated, thereby exerting less influence on neuronal message passing and the prepotent predictions (i.e., actions) to respond. In effect, the brain optimally would ignore the change (i.e., action update) signal by treating it as imprecise. Weak (Bayes-optimal) rSMG→rAI connections would mediate this uncertainty or imprecision. The subsequent message passing may be accomplished by Von Economo neurons (Evrard et al., 2012, 2014) communicating the rAI with the right ACC which was not modeled in this work. This speculation is consistent with the proposal that the right ACC encodes Bayesian surprise (Ide et al., 2013) because imprecise sensory prediction errors that are not attenuated at the level of the rAI would be attenuated at the level of the right ACC.

## Author Contributions

RL designed the study, analyzed data, wrote the manuscript. FJP analyzed data, wrote the manuscript. CM performed the study, wrote the manuscript. JLG-M wrote the manuscript.

## Conflict of Interest Statement

The authors declare that the research was conducted in the absence of any commercial or financial relationships that could be construed as a potential conflict of interest.
